# Supplementary material for: Long-term memory CD8+ T cells specific for SARS-CoV-2 in individuals who received the BNT162b2 mRNA vaccine
Source: Nat Commun. 2022 Sep 6;13:5251. doi: 10.1038/s41467-022-32989-4 (PMC9447987; doi:10.1038/s41467-022-32989-4)
Supplement: Supplementary file 1 — Supplementary Information [file 41467_2022_32989_MOESM1_ESM.pdf]

Supplementary Information

Long-term memory CD8+ T cells specific for SARS-CoV-2 in individuals who received the BNT162b2 mRNA vaccine

Nozomi Kuse[1], Yu Zhang[1], Takayuki Chikata[1], Hung The Nguyen[1], Shinichi Oka[1,2], Hiroyuki Gatanaga[1,2] and Masafumi Takiguchi[1]

1 Division of International Collaboration Research and Tokyo laboratory, Joint Research Center for Human Retrovirus Infection, Kumamoto University, Kumamoto/Tokyo, Japan

2 AIDS Clinical Center, National Center for Global Health and Medicine, Tokyo, Japan

Contents:

Supplementary Figure 1, 2, 3, 4
Supplementary Table 1, 2, 3, 4, 5
Supplementary References

Supplementary Figure 1

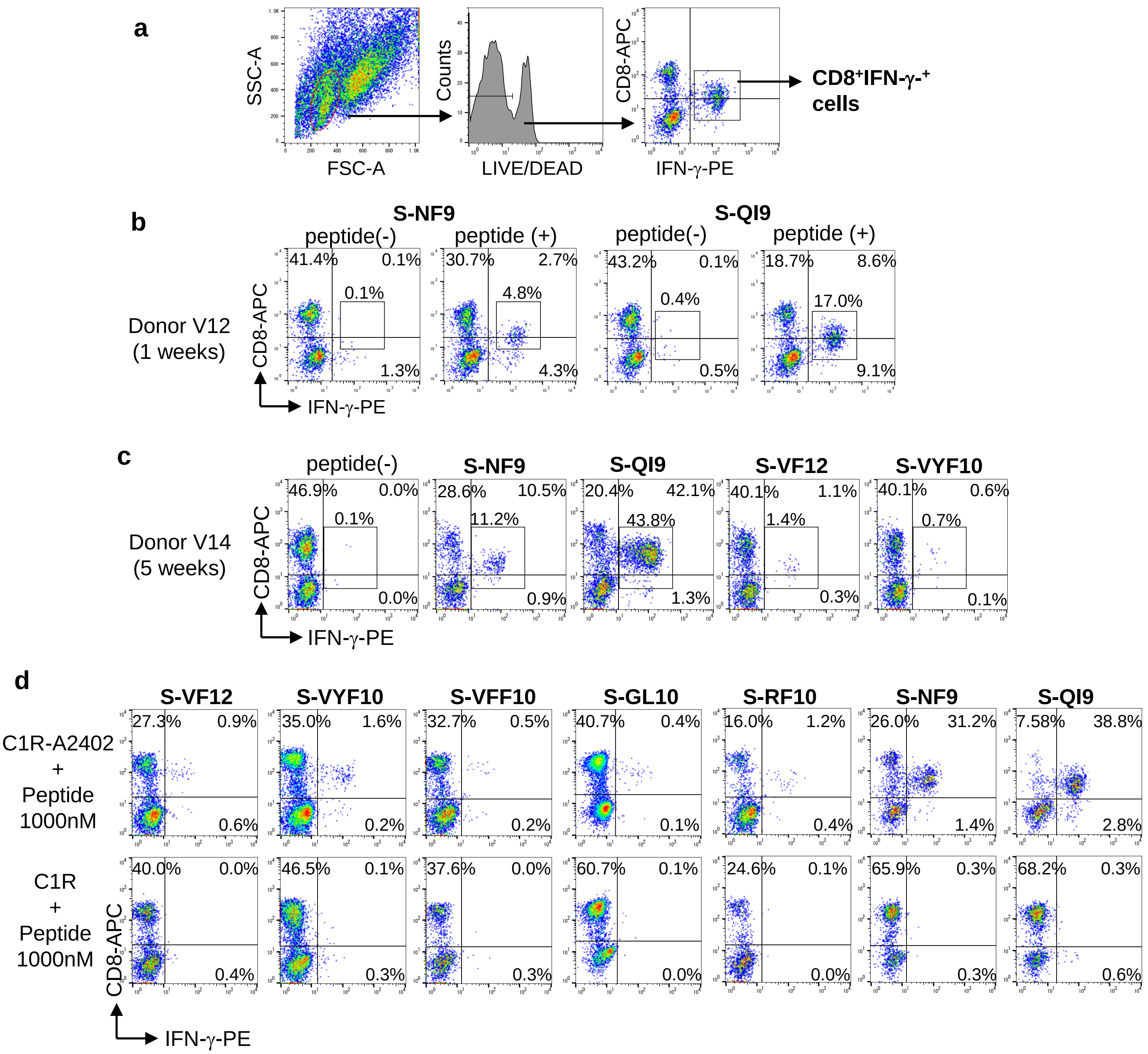

Supplementary Figure 1. IFN-γ⁺ cells among CD8 T cells in culture bulk T cells

(a) Flow cytometry strategy for detecting IFN-γ⁺ cells among CD8 T cells in Figures 1, 3, 5a, 5d, 6a, 7a and 7b. Representative flow cytometry dot plot staining of IFN-γ⁺ cells among CD8 T cells in Figure 1e **(b)**, Figure 1f (**c**), Figure 3 **(d)**.

Supplementary Figure 2

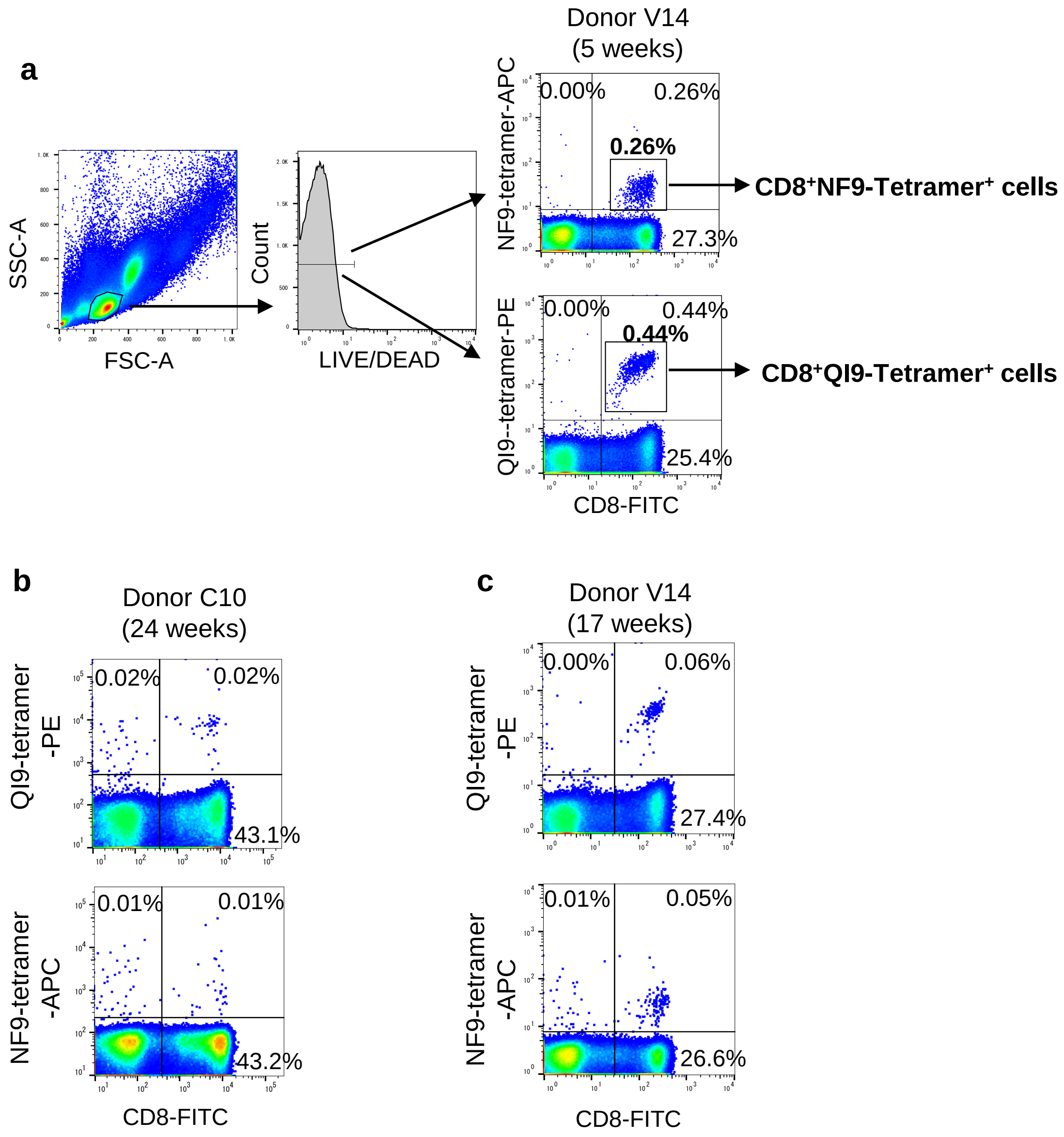

Supplementary Figure 2. HLA-A*24:02-restricted T cells specific for 2 dominant epitopes

(a) Flow cytometry strategy for detecting HLA-A*24:02-QI9 tetramer^{+} and HLA-A*24:02-NF9 tetramer^{+} cells among CD8 T cells in in Figures 2, 5b, 5c, 6b. Representative flow cytometry dot plot staining of tetramer^{+} cells among CD8 T cells in Figure 2e **(a)**, Figure 5b and Figure 5c (**b**), Figure 6b **(c)**.

Supplementary Figure 3

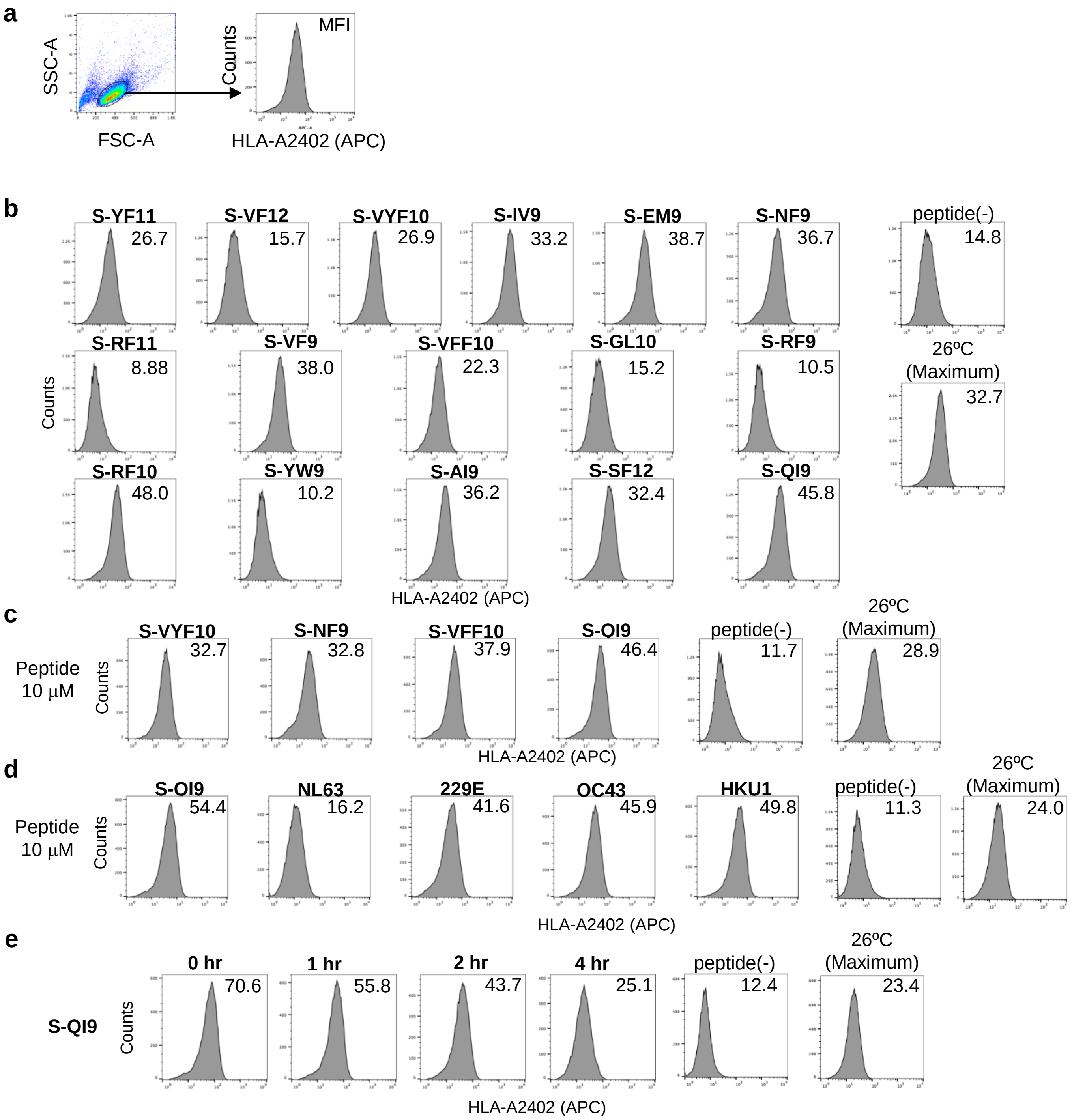

Supplementary Figure 3. Expression level of HLA-A*24:02 on RMA-S cell.

Representative plot show the gating strategy for Figure 4, 7c and 7d **(a)**. Representative flow cytometry staining of expression level of HLA-A2402 on RMA-S-A2402 cell in Figure 4a and Figure 4b **(b)**, Figure 4c **(c)**, Figure 7c **(d)** and Figure 7d **(e)**.

Supplementary Figure 4

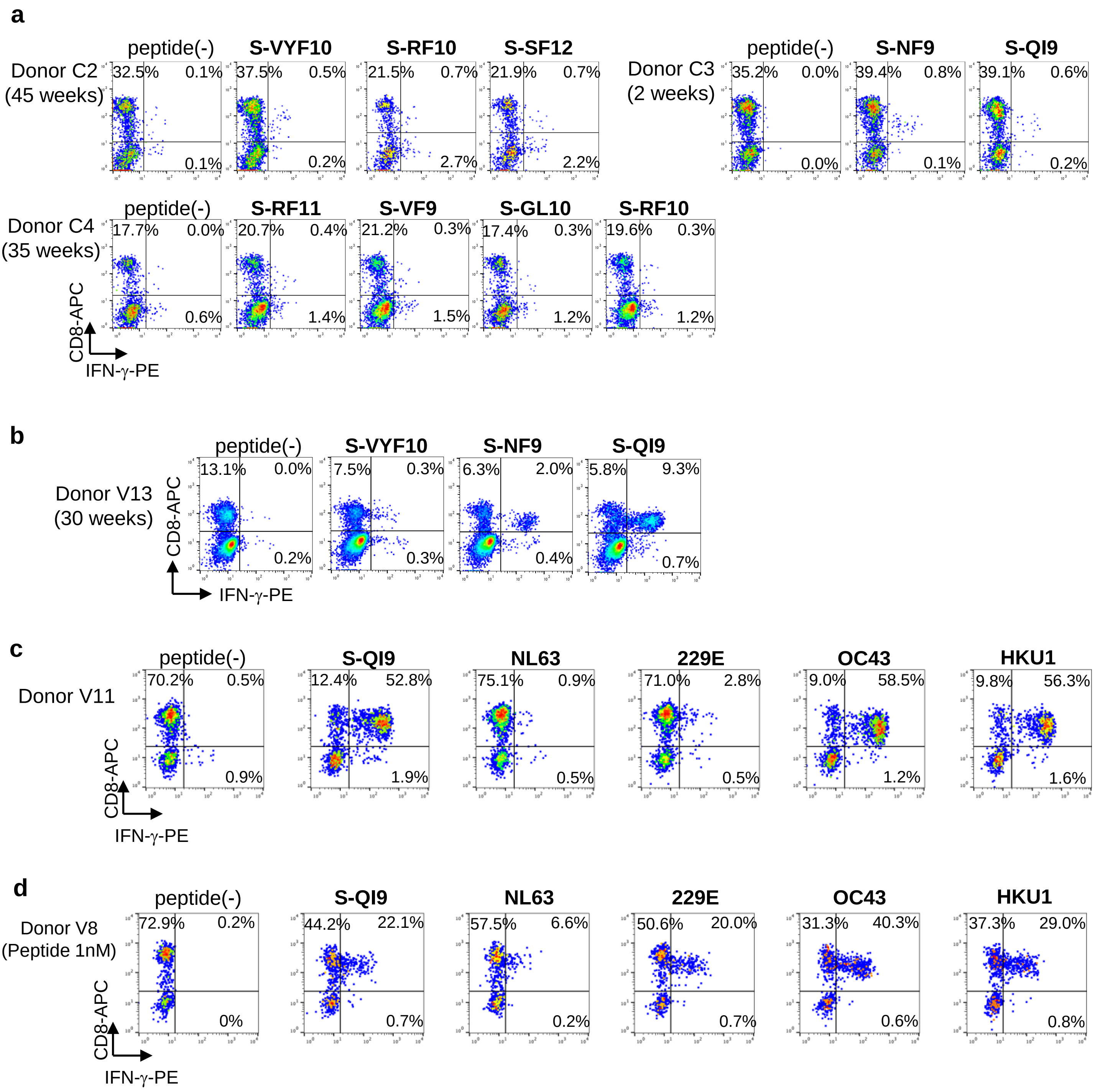

Supplementary Figure 4. Induction of memory CD8$^+$ T cells specific for immunodominant epitopes

Representative flow cytometry dot plot staining of IFN-γ$^+$ cells among CD8 T cells in Figure 5a and Figure 5d **(a)**, Figure 6a **(b)**, Figure 7a **(c)** and 7b **(d)**.

Supplementary Table 1. Donors in this study

ID	Age (years)	sex	S-IgG* (SU/ml)	N-IgG* (SU/ml)	HLA A allele		HLA B allele		HLA C allele	
V1	51	Female	46.2	0	A*02:07	A*24:02	B*46:01	B*52:01	C*01:02	C*12:02
V2	54	Female	41.5	0	A*02:01	A*24:02	B*51:01	B*07:02	C*14:02	C*07:02
V3	53	Male	97.4	0	A*24:02	A*24:02	B*35:01	B*40:02	C*01:02	C*15:02
V4	59	Male	109.4	0.2	A*24:02	A*26:01	B*15:18	B*40:02	C*08:01	C*03:04
V5	63	Male	33.6	0	A*02:01	A*24:02	B*35:01	B*51:01	C*01:02	C*03:03
V6	54	Male	114.9	0	A*02:07	A*24:02	B*46:01	B*55:10	C*01:02	C*01:02
V7	48	Male	165.3	0	A*11:01	A*24:02	B*07:02	B*15:02	C*07:02	C*08:01
V8	47	Male	161.2	0	A*11:01	A*24:02	B*40:06	B*48:01	C*08:01	C*08:01
V9	40	Male	76.6	0	A*24:02	A*31:01	B*35:01	B*52:01	C*04:01	C*12:02
V10	68	Male	471.9	0	A*11:01	A*24:02	B*52:01	B*52:01	C*12:02	C*14:02
V11	51	Male	474.2	0.1	A*24:02	A*24:20	B*40:02	B*52:01	C*01:02	C*12:02
V12	38	Female	558.6	0.1	A*01:01	A*24:02	B*07:02	B*40:01	C*03:04	C*07:02
V13	43	Male	39.5	0.2	A*24:02	A*24:02	B*52:01	B*52:01	C*12:02	C*12:02
V14	33	Female	283.2	0.1	A*11:01	A*24:02	B*51:01	B*51:01	C*14:02	C*14:02
V15	26	Male	334.1	0.1	A*11:01	A*24:02	B*07:02	B*40:01	C*04:01	C*07:02
V16	53	Male	80.3	0.1	A*24:02	A*31:01	B*40:03	B*54:01	C*01:02	C*03:04
V17	50	Female	102.2	0.1	A*11:01	A*24:02	B*52:01	B*54:01	C*01:02	C*12:02

*Anti-Spike IgG (S-IgG) and Anti-Nucleocapsid IgG (N-IgG) in serum were measured 2-4 weeks after the second injection of the BNT162b2 vaccine.

Supplementary Table 2. List of published HLA-A*24:02-restricted epitopes and method of indetification in previous studies.

Code	Peptide Pool Number	Name	Sequence	Position in Spike		Type of Assay							Donor type	Donor type	Reference
				Start	End	ex vivo Elispot	ex vivo ICS	ex vivo Tetramer	ex vivo AIM assay	ex vivo ELISA	Elispot with culture	ICS with culture			
1	1	S-YF11	YYVGYLQPRTF	265	275				●				convalescent		20
2	1	S-VF12	VYDPLQPELDSF	1137	1148				●				convalescent		20
3	1	S-VYF10	VYSSANNCTF	159	168				●				convalescent		20
4	1	S-IV9	IYQTSNFRV	312	320				●				convalescent		20
						●							convalescent		1*
5	1	S-EM9	EYVSQPFLM	169	177				●				convalescent		20
						●							convalescent		1*
6	1	S-NF9	NYNYLYRLF	448	456		●						convalescent		51
									●				convalescent		20
						●							convalescent		1*
								●				●	convalescent		52
								●					convalescent		54
								●					acute		2*
								●					vaccinated		8
												●	convalescent		59
								●					convalescent	acute	55
7	2	S-RF11	RFPNITNLCPF	328	338				●				convalescent		20
8	2	S-VF9	VFKNIDGYF	193	201				●				convalescent		20
						●							convalescent		1*
								●				●	convalescent		52
9	2	S-VFF10	VFVSNGTHWF	1094	1103				●				convalescent		20
10	2	S-GL10	GYLQPRTFLL	268	277				●				convalescent		20
11	2	S-RF9	RFDNPVLPF	78	86				●				convalescent		20
12	3	S-RF10	RVYSTGSNVF	634	643				●				convalescent		20
								●					unexposed	convalescent	3*
13	3	S-YW9	YYHKNNKSW	144	152				●				convalescent		20
14	3	S-AI9	AYSNNSIAI	706	714				●				convalescent		20
15	3	S-SF12	SFPQSAPHGVVF	1051	1062				●				convalescent		20
16	3	S-QI9	QYIKWPWYI	1208	1216		●						convalescent		51
									●				convalescent		20
										●			convalescent		56
											●		unexposed	convalescent	53
						●							convalescent		4*
								●				●	unexposed	convalescent	52
								●					convalescent		54
								●					acute		2*
								●					vaccinated		8
								●					unexposed	convalescent	5*
												●	unexposed		60
								●					convalescent	acute	55

* Supplementary references

Supplementary Table 3. COVID-19 Convalescent individuals

ID	Age (years)	sex	PCR-confirmed diagnosis (month/date/year)	PBMCs sampling (month/date/year)	HLA A allele		HLA B allele		HLA C allele	
C1	29	Male	03/23/20	02/03/21	A*02:06	A*24:02	B*07:02	B*39:01	C*07:02	-
C2	50	Male	01/24/21	02/10/21	A*24:02	A*26:03	B*35:01	B*54:01	C*01:02	C*03:03
C3	37	Male	07/06/20	08/03/21	A*02:07	A*24:02	B*15:07	B*44:03	C*01:02	C*14:03
C4	54	Male	08/08/20	05/25/21	A*02:06	A*24:02	B*52:01	B*55:02	C*01:02	C*12:02
C5	31	Male	01/14/21	06/03/21	A*24:02	A*26:02	B*52:01	B*54:01	C*01:02	C*12:02
C6	38	Male	01/06/21	06/07/21	A*24:02	A*33:03	B*15:35	B*58:01	C*03:02	C*07:02
C7	42	Male	07/14/20	06/14/21	A*02:06	A*24:02	B*07:02	B*40:06	C*07:02	C*08:01
C8	68	Male	01/15/21	06/17/21	A*24:02	-	B*35:01	B*51:01	C*03:03	C*14:02
C9	49	Male	12/28/20	06/18/21	A*02:07	A*24:02	B*40:02	B*51:01	C*14:02	C*15:02
C10	50	Male	01/27/21	07/16/21	A*24:02	-	B*40:01	B*52:01	C*12:02	C*15:02

Supplementary Table 4. Amino acid sequences of S-NF9 and S-QI9 epitopes among SARS-CoV-2 viruses

WHO	PANGO lineage	Amino acid position																	
		S-NF9*									S-QI9*								
		448	449	450	451	452	453	454	455	456	1208	1209	1210	1211	1212	1213	1214	1215	1216
		N	Y	N	Y	L	Y	R	L	F	Q	Y	I	K	W	P	W	Y	I
Alpha	B.1.1.7	N	Y	N	Y	L	Y	R	L	F	Q	Y	I	K	W	P	W	Y	I
Beta	B.1.351	N	Y	N	Y	L	Y	R	L	F	Q	Y	I	K	W	P	W	Y	I
Gamma	P.1	N	Y	N	Y	L	Y	R	L	F	Q	Y	I	K	W	P	W	Y	I
Delta	B.1.617.2	N	Y	N	Y	**R**	Y	R	L	F	Q	Y	I	K	W	P	W	Y	I
Omicron (BA.1)	B.1.1.529	N	Y	N	Y	L	Y	R	L	F	Q	Y	I	K	W	P	W	Y	I
Omicron (BA.5)	B.1.1.529	N	Y	N	Y	**R**	Y	R	L	F	Q	Y	I	K	W	P	W	Y	I

*Reference is Wuhan- Hu-1 sequence

Supplementary Table 5. Antibodies for flow cytometry

Antibody/Reagent	Catalogue No.	Company	Dilution
PE anti-human IFN-γ Antibody (Clone 4S.B3)	502509	BioLegend	1:100
APC anti-human CD8a Antibody(Clone HIT8a)	300912	BioLegend	1:100
Monoclonal Mouse Anti-Human CD8/FITC(Clone DK25)	F0765	DAKO	1:50
Alexa Fluor® 647 AffiniPure Sheep Anti-Mouse IgG (H+L)	515-605-003	Jackson ImmunoResearch	1:100
LIVE/DEAD Fixable Near-IR Dead Cell Stain Kit	L34975	Invitrogen	1:500
Streptavidin, R-Phycoerythrin conjugate	S866	Invitrogen	1.7μl in 10μg of monomer complexes
Streptavidin, Allophycocyanin, crosslinked, conjugate	S868	Invitrogen	1.7μl in 10μg of monomer complexes

Supplementary References.

1. Hu C, et al. Identification of cross-reactive CD8(+) T cell receptors with high functional avidity to a SARS-CoV-2 immunodominant epitope and its natural mutant variants. Genes Dis 9, 216-229 (2022).

2. Gangaev A, et al. Identification and characterization of a SARS-CoV-2 specific CD8(+) T cell response with immunodominant features. Nat Commun 12, 2593 (2021).

3. Saini SK, et al. SARS-CoV-2 genome-wide T cell epitope mapping reveals immunodominance and substantial CD8(+) T cell activation in COVID-19 patients. Sci Immunol 6, (2021).

4. Bilich T, et al. T cell and antibody kinetics delineate SARS-CoV-2 peptides mediating long-term immune responses in COVID-19 convalescent individuals. Sci Transl Med 13, (2021).

5. Nguyen THO, et al. CD8(+) T cells specific for an immunodominant SARS-CoV-2 nucleocapsid epitope display high naive precursor frequency and TCR promiscuity. Immunity 54, 1066-1082 e1065 (2021).
