# Supplementary material for: Long-term memory CD8+ T cells specific for SARS-CoV-2 in individuals who received the BNT162b2 mRNA vaccine
Source: Nat Commun. 2022 Sep 6;13:5251. doi: 10.1038/s41467-022-32989-4 (PMC9447987; doi:10.1038/s41467-022-32989-4)
Supplement: Supplementary file 2 — Reporting Summary [file 41467_2022_32989_MOESM2_ESM.pdf]

nature portfolio

Corresponding author(s): Masafumi Takiguchi

Last updated by author(s): Aug 10, 2022

# Reporting Summary

Nature Portfolio wishes to improve the reproducibility of the work that we publish. This form provides structure for consistency and transparency in reporting. For further information on Nature Portfolio policies, see our Editorial Policies and the Editorial Policy Checklist.

## Statistics

For all statistical analyses, confirm that the following items are present in the figure legend, table legend, main text, or Methods section.

| n/a | Confirmed | |
|---|---|---|
| ☐ | ☒ | The exact sample size ($n$) for each experimental group/condition, given as a discrete number and unit of measurement |
| ☐ | ☒ | A statement on whether measurements were taken from distinct samples or whether the same sample was measured repeatedly |
| ☐ | ☒ | The statistical test(s) used AND whether they are one- or two-sided<br>*Only common tests should be described solely by name; describe more complex techniques in the Methods section.* |
| ☒ | ☐ | A description of all covariates tested |
| ☒ | ☐ | A description of any assumptions or corrections, such as tests of normality and adjustment for multiple comparisons |
| ☐ | ☒ | A full description of the statistical parameters including central tendency (e.g. means) or other basic estimates (e.g. regression coefficient) AND variation (e.g. standard deviation) or associated estimates of uncertainty (e.g. confidence intervals) |
| ☐ | ☒ | For null hypothesis testing, the test statistic (e.g. $F$, $t$, $r$) with confidence intervals, effect sizes, degrees of freedom and $P$ value noted<br>*Give P values as exact values whenever suitable.* |
| ☒ | ☐ | For Bayesian analysis, information on the choice of priors and Markov chain Monte Carlo settings |
| ☒ | ☐ | For hierarchical and complex designs, identification of the appropriate level for tests and full reporting of outcomes |
| ☐ | ☒ | Estimates of effect sizes (e.g. Cohen's $d$, Pearson's $r$), indicating how they were calculated |

*Our web collection on statistics for biologists contains articles on many of the points above.*

## Software and code

Policy information about availability of computer code

| | |
|---|---|
| Data collection | FACS data acquisition: FACSDiva software v9.0(BD). |
| Data analysis | Flowcytometry analyses were performed using Flowjo v10(BD) .<br><br>All Statistics were performed using Prism v8.0 software (GraphPad). |

For manuscripts utilizing custom algorithms or software that are central to the research but not yet described in published literature, software must be made available to editors and reviewers. We strongly encourage code deposition in a community repository (e.g. GitHub). See the Nature Portfolio guidelines for submitting code & software for further information.

## Data

Policy information about availability of data

All manuscripts must include a data availability statement. This statement should provide the following information, where applicable:

- Accession codes, unique identifiers, or web links for publicly available datasets
- A description of any restrictions on data availability
- For clinical datasets or third party data, please ensure that the statement adheres to our policy

All data are available in the main article and its supplementary information or from the corresponding author upon reasonable request. Source data are provided with this paper.

## Human research participants

Policy information about studies involving human research participants and Sex and Gender in Research.

| | |
|---|---|
| Reporting on sex and gender | The data participants' biological sex are presented in supplemental information. |
| Population characteristics | The details of the individuals were shown in supplemental table. HLA types of these individuals were determined by standard sequence-based genotyping. |
| Recruitment | Participants who received two doses of the Pfizer-BioNTech BNT162b2 mRNA vaccine were recruited through Kumamoto University in Japan. Participants who had been infected with SARAS-CoV-2 were recruited through the National Center for Global Health and Medicine in Japan. We estimate that there were no self-selection bias or any other bias in the recruitment of the volunteers in the present study. |
| Ethics oversight | This study was approved by the Ethics Committee of Kumamoto University, Japan and the National Center for Global Health and Medicine, Japan. Informed consent was obtained from all individuals according to the Declaration of Helsinki. |

Note that full information on the approval of the study protocol must also be provided in the manuscript.

# Field-specific reporting

Please select the one below that is the best fit for your research. If you are not sure, read the appropriate sections before making your selection.

- [x] Life sciences
- [ ] Behavioural & social sciences
- [ ] Ecological, evolutionary & environmental sciences

For a reference copy of the document with all sections, see nature.com/documents/nr-reporting-summary-flat.pdf

# Life sciences study design

All studies must disclose on these points even when the disclosure is negative.

| | |
|---|---|
| Sample size | Seventeen HLA-A*24:02+ individuals who received two doses of the Pfizer-BioNTech BNT162b2 mRNA vaccine with three weeks interval and ten HLA-A*24:02+ individuals who had been infected with SARAS-CoV-2were recruited. Sample sizes were based on available sample sets. |
| Data exclusions | No data was excluded. |
| Replication | All attempts at replication were successful for experiments using cell lines. The data shown in Fig4C and Fig7C were obtained from two independent experiments. The experiments in Fig3 except that using S-VF12 peptide were repeated twice. Due to limited sample availability, other experiment using primary cells isolated from vaccinated donors and convalescent COVID-19 donors were not run in duplicate and not repeated. |
| Randomization | The study subjects are part of an observational cohort without therapeutic intervention. Randomization was therefore not possible for this study. |
| Blinding | Blinding regarding group assignments was not implemented in this study, since no subjective variables were measured. |

# Reporting for specific materials, systems and methods

We require information from authors about some types of materials, experimental systems and methods used in many studies. Here, indicate whether each material, system or method listed is relevant to your study. If you are not sure if a list item applies to your research, read the appropriate section before selecting a response.

## Materials & experimental systems

| n/a | Involved in the study | |
|---|---|---|
| [ ] | [x] | Antibodies |
| [ ] | [x] | Eukaryotic cell lines |
| [x] | [ ] | Palaeontology and archaeology |
| [x] | [ ] | Animals and other organisms |
| [x] | [ ] | Clinical data |
| [x] | [ ] | Dual use research of concern |

## Methods

| n/a | Involved in the study | |
|---|---|---|
| [x] | [ ] | ChIP-seq |
| [ ] | [x] | Flow cytometry |
| [x] | [ ] | MRI-based neuroimaging |

## Antibodies

Antibodies used

Information (supplier name, catalog number, clone name, and dilution) of all antibodies used in the present study are available in the Supplemental table.
・PE anti-human IFN-γ Antibody (Clone 4S.B3), BioLegend, Cat# 502509, 1:100 dilution
・APC anti-human CD8a Antibody(Clone HIT8a), BioLegend, Cat# 300912, 1:100 dilution
・Monoclonal Mouse Anti-Human CD8/FITC(Clone DK25), DAKO, Cat# F0765, 1:50 dilution
・Alexa Fluor® 647 AffiniPure Sheep Anti-Mouse IgG (H+L), Jackson ImmunoResearch, Cat# 515-605-003, 1:100 dilution
・LIVE/DEAD Fixable Near-IR Dead Cell Stain Kit, for 633 or 635 nm excitation, Invitrogen, Cat# L34975, 1:500 dilution
・Streptavidin, R-Phycoerythrin Conjugate, Invitrogen, Cat# S866, 1.7ml in 10mg of monomer complexes
・Streptavidin, Allophycocyanin, crosslinked, conjugate, Invitrogen, Cat# S868, 1.7ml in10mg of monomer complexes

・Mouse anti-human HLA-A24 monoclonal antibody (clone. A11.1M, culture supernatant of hybridoma cell line A11.1M) was generated by our lab.
・HLA-class I-peptide tetrameric complexes (HLA-A2402-NF9 and HLA-A2402-QI9-tetramers) were synthesized by our lab.
The detailed information was described in the Methods.

Validation

・Mouse anti-human HLA-A24 monoclonal antibody (clone. A11.1M) was prepared by collecting culture supernatant of hybridoma cell line A11.1M and used as primary antibody for HLA stabilization assay.
・HLA-class I-peptide tetrameric complexes (HLA-A2402-NF9 and HLA-A2402-QI9-tetramers) were synthesized by our lab and used for tetramer staining assay.

These antibodies and reagents were validated and dilution optimized using positive cells (RMA-S-A2402 cells or PBMC) before performance of the study.

Other antibodies were validated by manufacturers; this information is provided on the manufacturers' website and product information datasheets as follow:

・IFN-γ PE (Clone 4S.B3; cat. 502509), BioLegend
https://www.biolegend.com/ja-jp/products/pe-anti-human-ifn-gamma-antibody-1011

・CD8 APC (Clone HIT8a; cat. 300912), BioLegend
https://www.biolegend.com/en-us/search-results/apc-anti-human-cd8a-antibody-759?Clone=HIT8a

・CD8 FITC (Clone DK25; cat. F0765), DAKO
https://www.citeab.com/antibodies/3382948-f0765-cd8

・Sheep Anti-Mouse IgG (H+L) Alexa Fluor 647-AffiniPure (cat. 515-605-003), Jackson ImmunoResearch.
https://www.jacksonimmuno.com/catalog/products/515-605-003

・LIVE/DEAD Fixable Near-IR Dead Cell Stain Kit, for 633 or 635 nm excitation (cat. L34975), Invitrogen.
https://www.thermofisher.com/order/catalog/product/L34975

・Streptavidin, R-Phycoerythrin Conjugate (cat. S866), Invitrogen
https://www.thermofisher.com/order/catalog/product/S866

・Streptavidin, Allophycocyanin, crosslinked, conjugate (cat. S868), Invitrogen
https://www.thermofisher.com/order/catalog/product/S868?SID=srch-hj-S868

## Eukaryotic cell lines

Policy information about cell lines and Sex and Gender in Research

Cell line source(s)

C1R and RMA-S were obtained from the ATCC.

Authentication

C1R and RMA-S were not authenticated.

Mycoplasma contamination

C1R and RMA-S were tested for mycoplasma contamination and were found negative.

Commonly misidentified lines
(See ICLAC register)

No commonly misidentified cell lines were used in this study.

## Flow Cytometry

### Plots

Confirm that:

- [x] The axis labels state the marker and fluorochrome used (e.g. CD4-FITC).
- [x] The axis scales are clearly visible. Include numbers along axes only for bottom left plot of group (a 'group' is an analysis of identical markers).
- [x] All plots are contour plots with outliers or pseudocolor plots.
- [x] A numerical value for number of cells or percentage (with statistics) is provided.

### Methodology

| | |
|---|---|
| Sample preparation | Peripheral blood mononuclear cells (PBMC) were isolated from blood via density gradient centrifugation on Ficoll-Paque PLUS (GE Healthcare Life Sciences) and were cryopreserved. Thawed PBMCs were stimulated with peptide and then culture for 2 weeks for induction bulk T cells or used for tetramer staining assay. The bulk T cells were used for intracellular cytokine staining assay. |
| Instrument | FACSCanto II (BD). |
| Software | FlowJo v10 software (BD) |
| Cell population abundance | All cell population tested were abundant (10, 000events collected) |
| Gating strategy | Appropriate gating was applied in the FSC/SSC window. Dead cells were removed by viability staining. Additional gating was applied for the analysis of specific cell subpopulations. |

- [x] Tick this box to confirm that a figure exemplifying the gating strategy is provided in the Supplementary Information.
